# Dietary calcium intake in a cohort of individuals evaluated for low bone mineral density: a multicenter Italian study

**DOI:** 10.1007/s40520-021-01856-5

**Published:** 2021-04-28

**Authors:** Elisa Cairoli, Carmen Aresta, Luca Giovanelli, Cristina Eller-Vainicher, Silvia Migliaccio, Sandro Giannini, Andrea Giusti, Claudio Marcocci, Stefano Gonnelli, Gian Carlo Isaia, Maurizio Rossini, Iacopo Chiodini, Marco Di Stefano, the Italian Society for Osteoporosis, Mineral Metabolism, Skeletal Diseases (SIOMMMS), Valter Galmarini, Giovanni Passeri, Fabio Di Salvo, Giangiacomo Osella, Francesco Tripodi, Roberto Valenti, Gloria Bonaccorsi, Gilberta Giacchetti, Flavia Pugliese, Andrea Casabella, Bruno Seriolo, Antonio Giovanni Emilio Masala, Pileri Piera Veronica, Letizia Maninetti, Alessio De Santis, Alfredo Bardoscia, Alice Parma, Paolo Caso, Monica Mazza

**Affiliations:** 1 Unit for Bone Metabolism Disease and Diabetes and Lab of Endocrine and Metabolic Research, Istituto Auxologico Italiano IRCCS, Via Magnasco 2, 20149 Milan, Italy; 2 Department of Pathophysiology and Transplantation, University of Milan, Milan, Italy; 3 Department of Medical Biotechnology and Translational Medicine, University of Milan, Milan, Italy; 4 Unit of Endocrinology, Fondazione IRCCS Ospedale Maggiore Policlinico, Milan, Italy; 5 Department of Movement, Human and Health Sciences, University of Rome “Foro Italico”, Rome, Italy; 6 Department of Medicine, Medical Clinic 1, University of Padua, Padua, Italy; 7 Unit of Rheumatology, ASL3-Liguria Region, Genoa, Italy; 8 Department of Clinical and Experimental Medicine, University of Pisa, Pisa, Italy; 9 Department of Medicine, Surgery and Neuroscience, Le Scotte University Hospital, University of Siena, Siena, Italy; 10 Department of Medical Science, Section of Gerontology and Bone Metabolic Disease, University of Turin, Turin, Italy; 11 Department of Medicine, Rheumatology Unit, University of Verona, Verona, Italy

**Keywords:** Dietary calcium intake, Osteoporosis, Fractures, Bone mineral density

## Abstract

**Background:**

A low calcium intake is a well-known factor that influences the bone mineral density (BMD) maintenance. In the presence of inadequate calcium intake, secondary hyperparathyroidism develops, leading to an increased bone turnover and fracture risk.

**Aims:**

To assess the dietary calcium intake in relation with osteoporosis and fragility fracture in a cohort of Italian individuals evaluated for low BMD.

**Methods:**

A 7-day food-frequency questionnaire was administered to 1793 individuals, who were consecutively referred at the Centers of the Italian Society for Osteoporosis, Mineral Metabolism and Skeletal Diseases (SIOMMMS) for low BMD.

**Results:**

In 30.3% and 20.9% of subjects, the calcium intake was inadequate (< 700 mg/day) and adequate (> 1200 mg/day), respectively. As compared with patients with adequate calcium intake, those with inadequate calcium intake were younger (65.5 ± 10.8 vs 63.9 ± 11.5 years, *p* = 0.03) and they more frequently reported adverse reactions to food (3.2% vs 7.2% *p* = 0.01) and previous major fragility fractures (20.8% vs 27.0%, *p* = 0.03). Patients with calcium intake < 700 mg/day showed a higher prevalence of diabetes mellitus, idiopathic hypercalciuria and food allergy/intolerance (8.1%, 5.1%, 7.2%, respectively) than patients with calcium intake > 700 mg/day (5.3%, 3.0%, 4.1%, respectively, *p* < 0.04 for all comparisons), also after adjusting for age, gender and body mass index. In 30.3% of fractured subjects, the calcium intake was < 700 mg/day.

**Discussion:**

In Italy, a low calcium intake is highly prevalent in individuals at risk for low BMD. Importantly, an inadequate calcium intake is highly prevalent even in patients with history of fragility fractures.

**Conclusions:**

Only about a fifth of patients being assessed for low BMD in an Italian SIOMMMS referral Centre have an adequate calcium intake.

## Introduction

Calcium intake is a well-known factor that influences the achievement of an adequate peak bone mass [1] and, subsequently, the maintenance of bone mass later in life. Indeed, in the presence of inadequate calcium intake, a negative calcium balance can develop. This frequently leads to metabolic alterations, such as secondary hyperparathyroidism, increased bone turnover and, eventually, may increase the fracture risk [2].

The calcium intake largely differs among countries according to age, sex, ethnics, cultures and socioeconomic status [3] and the national recommendations on calcium intake vary worldwide. In the adult population, the Recommended Dietary Allowance (RDA) of calcium is between 1000 and 1300 mg/day according to the US National Institutes of Health [4] and between 700 and 1000 mg/day according to the UK National Osteoporosis Society [5]. Both the Italian Society for Osteoporosis, Mineral Metabolism and Skeletal Diseases (SIOMMMS) and the Italian Society of Human Nutrition recommend a calcium intake above 1200 mg/day in postmenopausal women not on hormone replacement therapy and in men older than 60–65 years of age [6, 7].

The adequate daily calcium requirement is influenced by several other factors, such as age, comorbidities, and vitamin D levels, the latter being crucial for an adequate intestinal calcium absorption [8, 9]. Even due to the inter-individual variability of intestinal calcium absorption, the threshold of calcium intake below which the use of calcium supplements is indicated is largely unknown [9]. However, there is a general agreement that a dietary calcium intake below 700 mg/day requires calcium supplementation, while, in patients with low bone mineral density (BMD), a calcium intake above 1200 mg/day is adequate [7, 8, 10, 11]

Despite this unsolved issue, a recent systematic review found that dietary calcium intakes fall below the recommended levels in many areas of the world [12], including Italy [13]. This is a matter of concern for bone health, especially in populations at risk for low BMD, in whom an adequate calcium intake represents one of the first non-pharmacological interventions, which, in the presence of an adequate vitamin D status, has a positive effect on fracture risk [8–11].

This multicenter Italian cross-sectional observational study was aimed to assess in patients with possible low BMD: (i) the overall calcium intake and its relation with BMD and fragility fractures; (ii) the prevalence and characteristics of patients with a calcium intake so low that normally requires supplementation (i.e., < 700 mg/day); (iii) the prevalence and characteristics of patients with an adequate calcium intake (i.e., > 1200 mg/day).

## Patients and methods

### Patients

**Fig. 1:**
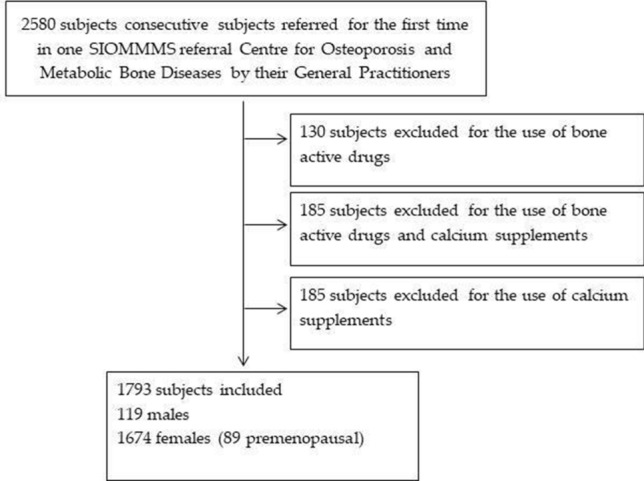
The inclusion protocol. Between November 2015 and June 2016, 1793 consecutive subjects referred in 1 SIOMMMS referral Centre for Osteoporosis and Metabolic Bone Diseases by their General Practitioners that agreed in participating in this study, were recruited. We excluded subjects reporting the intake of calcium supplements and/or bone active drugs because these treatments inevitably imply some kind of previous medical counselling about osteoporosis and the related importance of an adequate calcium intake and then estimated dietary calcium intake would have not reflected their usual dietary habits

The study has been carried out in 17 different SIOMMMS centers spread all over Italy (5 in Northern Italy, 6 in Middle Italy, 6 in Southern Italy). Between November 2015 and June 2016, 1793 consecutive subjects who were referred for the first time to an Italian SIOMMMS referral Centre for Osteoporosis and Metabolic Bone Diseases by their General Practitioners and agreed in participating in this study, were recruited. We excluded subjects reporting the intake of calcium supplements and/or bone active drugs. Indeed, since these treatments inevitably imply some kind of previous medical counselling about osteoporosis and the importance of an adequate calcium intake, the estimated dietary calcium intake would not have represented the usual dietary habits. The inclusion protocol is reported in Fig. 1**.**

### Methods

In all individuals, height (expressed in meters) and weight (expressed in kilograms) were measured, by calibrated scales and stadiometer, respectively, and body mass index (BMI, kg/m^2^) was calculated.

The dietary calcium intake, expressed as mg/day, was assessed using a specific questionnaire. In particular, usual calcium intake coming from some selected calcium-rich foods was estimated by a 7-day food frequency questionnaire derived for the International Osteoporosis Foundation (IOF) Calcium Calculator [14] after simplification to specifically focus on calcium intake only and in accordance to the ordinary Italian alimentary habits.

Portion sizes were quantified by means of household measures (slices, cups, glasses). The time interval between the questionnaire and BMD measurement was ± 3 months. The questionnaire has been always administrated by investigators blinded to the clinical and BMD data of the patients. Furthermore, in 200 consecutive patients, the questionnaire has been administrated simultaneously by 2 different investigators and the interrater reliability between the 2 investigators was satisfactory (*κ* = 0.85). The history of clinical fragility fractures (i.e., caused by low energy trauma, such as falling from a standing height or less) was investigated at consultation. Hip and vertebral fractures (major fragility fractures) were considered apart from the others (e.g., wrist, ribs and proximal humerus) and were verified by consulting medical records. At variance, the presence of previous fragility fractures other than hip and vertebral fractures was ascertained by self-report and no additional validation of this information was conducted. No spinal radiograph was performed for assessing the presence of morphometric vertebral fractures. If nephrolithiasis was reported by the patients, the medical records (ultrasound and/or abdominal radiograph) were reviewed.

Information about BMD, measured by Dual-energy X-ray Absorptiometry (DXA) using reliable densitometers (QDR 4500 or Horizon or Discovery, Hologic, USA; Lunar Prodigy, iDXA, DPX-IQ General Electric, USA) at lumbar spine, total femur and femoral neck and expressed as standard deviation units in relation to the young (T-score) and age-matched (Z-score) reference healthy population, were collected. In all patients, DXA was performed within 12 months before the enrolment and only one BMD determination has been considered. The DXA machines have not been calibrated across participating center and DXA scans had been carried out according to the Italian Ministry of Health recommendations [15]. As our sample included postmenopausal females, premenopausal females and men younger than 50, we used the term “low BMD” in the presence of T-score at any site ≤  − 2.5 for postmenopausal women and men older than 50 [16] or in the presence of Z-score at any site <  − 2.0 for premenopausal women and men younger than 50 [17].

Demographic and clinical data were anonymously collected and the presence of following comorbidities was recorded: diabetes mellitus, endogenous or iatrogenic hypercortisolism, rheumatoid arthritis (AR), idiopathic hypercalciuria, primary hyperparathyroidism (PHPT), nephrolithiasis, adverse reaction to food (including food allergy or intolerance, i.e., coeliac disease and lactose intolerance) [18], inflammatory bowel diseases (IBD) and chronic obstructive pulmonary disease (COPD). Clinical data were confirmed by the review of medical reports. No blood or additional instrumental tests were performed.

In accordance with the available literature [7], we considered a calcium intake < 700 mg/day as the threshold below which the dietary calcium intake is clearly inadequate and a supplementation is generally considered mandatory. On the contrary, we considered a calcium intake > 1200 mg/day as the threshold for defining an adequate calcium intake, although this threshold is suggested by SIOMMMS [6] specifically in men or premenopausal women. We decided to use this threshold for the sake of consistency in the data analysis, since, as postmenopausal females, even men and premenopausal females were sent to our outpatient Clinics for low BMD and/or fragility fracture and since they represented a minority of the entire cohort (*n* = 119 and *n* = 88, respectively).

This study was conducted according to the guidelines laid down in the Declaration of Helsinki and all procedures involving human subjects/patients were approved (November 15th, 2015) by the Ethical Committee of each SIOMMMS center and all subjects gave their written informed consent before participating in the study.

### Statistical analysis

For the sample size calculation, the software PS Power and Sample Size Calculations (Version 3.0, January 2009, http://biostat.mc.vanderbilt.edu/PowerSampleSize) has been used.

In accordance with the first study aim, to have a meaningful index for evaluating the sample size, we decided to consider the possible difference in calcium intake between patients with and without major fragility fractures. In a previous study reporting the daily calcium intake in subjects with and without vertebral fracture [19], the response within each subject group was normally distributed (standard deviation 300), the difference in daily calcium intake between fractured and not fractured patients was 60 mg/day and the prevalence of very low calcium intake was significantly different between patients with clinical vertebral fracture and those without clinical vertebral fracture. On the basis of these data, for the study to be adequately powered to disclose differences in calcium intake between fractured and not fractured subject, we needed at least 329 fractured subjects and 1316 not fractured (power 90%, type I error 5%). This figure is in keeping with data from a previous study on 311 post-menopausal female with low dietary calcium intake and 622 matched control subjects in which the prevalences of osteoporosis and of wrist fracture were significantly different between subjects with low calcium intake as compared with control subjects [20].

Statistical analysis was performed by SPSS version 21.0 statistical package (SPSS Inc, Chicago, IL). For each continuous variables, the normality of distribution was tested by the Kolmogorov–Smirnov test. Data were expressed as median (range) for non-normally distributed continuous variables or as mean ± standard deviation for normally distributed variables, and as absolute and relative frequencies for categorical variables. Continuous variables were compared using one-way Student t test or Mann–Whitney U test, as appropriate. Comparison of continuous variables among groups was performed using one-way ANOVA and Bonferroni post-hoc analysis as appropriate. Categorical variables were compared using χ^2^ or Fisher's Exact test, as appropriate.

The multivariate logistic regression analysis was performed to assess the association between the presence of either major fragility fractures or inadequate or adequate calcium intake (categorical-dependent variables) and age, gender, BMI and the variables which resulted to be statistically different from the comparisons either between patients with major fragility fractures and those without major fragility fractures or between patients with calcium intake < 700 mg/day and those with calcium intake ≥ 700 mg/day or between patients with calcium intake ≥ 1200 mg/day and those with calcium intake < 1200 mg/day.

*P* values < 0.05 were considered significant.

## Results

### Overall calcium intake and its relation with the clinical characteristics of the patients

Overall, 368 patients had a major fragility fracture, while 1425 subjects did not. Therefore the study was adequately powered to disclose differences in calcium intake between fractured and not fractured patients. The clinical characteristics of the whole cohort and the comparisons among individuals grouped according tertiles of dietary calcium intake are reported in Table 1. In the entire cohort the median (± standard error) calcium intake was 874.9 (± 4.5) mg/day, the 30.3% of the enrolled subjects showed a clearly inadequate calcium intake and in only 20.9% of subjects the calcium intake was adequate.

**Table 1:** Clinical characteristics of the whole cohort and the comparisons among individuals grouped according tertiles of dietary calcium intake

	All subjects (*n* = 1793)	I tertile (*n* = 598)	II tertile (*n* = 598)	III tertile (*n* = 597)
Daily calcium intake (mg/day)	874.9	< 723	723–1043	> 1043
Sex (females)	1674 (93.4)	567 (94.8)	556 (93.0)	551 (92.3)
Premenopausal females	88 (5.3)	41 (7.2)^**2**^	28 (5.0)	19 (3.4)
Age (years)	65.0 (25–97)	64.5 (25–97)^**2**^	65.0 (28–90)^**2**^	66.0 (27–94)
BMI (kg/m^2^)	24.6 (14.2–48.6)	25.1 (14.3–48.6)	24.2 (14.2–43.9)	24.3 (15.1–44.9)
Low BMD	778 (43.4)	253 (42.3)^**2**^	242 (40.5)^**2**^	283 (47.3)
Prevalence of calcium intake < 700 mg/day	544 (30.3)	544 (100)	0 (0.0)	0 (0.0)
Prevalence of calcium intake > 1200 mg/day	374 (20.9)	0 (0.0)	0 (0.0)	374 (100)
Major fragility fractures	368 (20.5)	119 (19.9)^**2**^	99 (16.6)^**1**^	150 (25.1)
Other fragility fractures	334 (18.6)	102 (17.1)	119 (19.9)	113 (18.9)
Low BMD and/or major fragility fractures	901 (503)	297 (49.7)^**2**^	279 (46.7)^**1**^	325 (54.4)
Diabetes Mellitus	110 (6.1)	47 (7.9)	32 (5.4)	31 (5.2)
Hypercortisolism (endogenous or exogenous)	137 (7.6)	44 (7.3)	48 (8.0)	45 (7.5)
RA	269 (15.0)	87 (14.5)	93 (15.6)	89 (14.9)
Idiopathic hypercalciuria	65 (3.6)	31 (5.2)^**2**^	18 (3.0)	16 (2.7)
PHPT	30 (1.7)	7 (1.2)	11 (1.8)	12 (2.0)
Nephrolitiasis	132 (7.4)	47 (7.9)	49 (8.2)	36 (6.0)
Adverse reaction to food	90 (5.0)	41 (6.9)^**2,3**^	25 (4.2)	24 (4.0)
IBD	65 (3.6)	27 (4.5)	16 (2.7)	22 (3.7)
COPD	71 (4.0)	22 (3.7)	24 (4.0)	25 (4.2)

Data are expressed as median values (range) or absolute number (percentage)

Other fragility fractures: wrist, ribs and proximal humerus fractures. I tertile: 68.3–723.7 mg/day; II tertile 725.0–1042, 9 mg/day; III tertile 1043.1–3534.4 mg/day; ^1^* p* < 0.005 vs tertile III; ^2^* p* < 0.05 vs tertile III; ^3^* p* < 0.05 vs tertile II

*BMI* body mass index, *RA* rheumatoid arthritis, *PHPT* primary hyperparathyroidism, *IBD* inflammatory bowel disease, *COPD* chronic obstructive pulmonary disease, *Low BMD* T-score at any site ≤  − 2.5 for postmenopausal women and men older than 50 or in the presence of Z-score at any site <  − 2.0 for premenopausal women and men < 50 years

The enrolled subjects were mainly females and were comparable among the different tertiles as far as gender, BMI and prevalence of the main comorbidities. Subjects in the lowest and intermediate tertiles were younger and showed a lower prevalence of low BMD and major fragility fractures than those in the highest tertile. Moreover, the prevalence of premenopausal females, idiopathic hypercalciuria and adverse reaction to food was higher in subjects in the lowest tertile than in those in the highest tertile of daily dietary calcium intake.

Three hundred 68 subjects reported a previous major fragility fracture, whereas the remaining 1425 did not, thus respecting the sample size calculation requested to guarantee the adequate power of the study. Patients included in the II tertiles show intermediate characteristics as compared with patients included in the I and III tertile as far as prevalence of patients with low BMD and/or fragility fractures.

The presence of a major fragility fracture was associated with calcium intake, age, male gender and low BMD regardless of menopausal status and BMI, by logistic regression analysis (Table 2).

**Table 2:** Significant independent predictors of the presence of a major fragility fracture (panel A) or of an inadequate calcium intake (panel B) or of an adequate calcium intake (panel C)

Panel A Major Fragility Fracture	OR	95%CI	*P*
Calcium Intake (1 mg/day increase)	1.01	1.01–1.02	0.034
Male Gender (presence)	1.81	1.15–2.87	0.011
Age (1 year increase)	1.07	1.06–1.083	0.0001
Low BMD (presence)	2.77	2.14–3.7	0.0001

Panel B Calcium Intake < 700 mg/day	OR	95%CI	*P*
Female Gender (presence)	1.58	1.01–2.47	0.047
Diabetes Mellitus (presence)	1.61	1.07–2.42	0.023
Food intolerance (presence)	1.82	1.18–2.82	0.007
Idiopathic hypercalciuria (presence)	1.73	1.04–2.89	0.035

Panel C Calcium Intake ≥ 1200 mg/day	OR	95%CI	*P*
BMI (1 kg/m^2^ decrease)	1.04	1.01–1.06	0.016
History of major fragility fracture (presence)	1.55	1.16–2.07	0.003
Previous diagnosis of PHPT (presence)	2.66	1.23–5.75	0.013
Nephrolithiasis (absence)	1.86	1.10–3.14	0.020

Major Fragility Fracture: hip and vertebral fractures; BMI: body mass index. OR: odds ratio; 95%CI: 95% confidence interval. PHPT: primary hyperparathyroidism. Low BMD: T-score at any site ≤  − 2.5 for postmenopausal women and men older than 50 or in the presence of Z-score at any site <  − 2.0 for premenopausal women and men < 50 years

The variables included in all models were age, gender, BMI and those variables which resulted to be statistically different in the comparisons either between patients with major fragility fractures and those without major fragility fractures (Panel A), or between patients with calcium intake < 700 mg/day and those with calcium intake ≥ 700 mg/day (Panel B), or between patients with calcium intake ≥ 1200 mg/day and those with calcium intake < 1200 mg/day (Panel C)

As compared with patients with adequate calcium intake (i.e., < 1200 mg/day), patients with inadequate calcium intake (< 700 mg/day) were younger and had higher BMI, prevalence of diabetes mellitus, nephrolithiasis and adverse reaction to food and lower prevalence of PHPT history (Table 3).

**Table 3:** Comparisons between subjects with calcium intake < 700 mg/day and > 1200 mg/day

	Calcium intake < 700 mg/day (*n* = 544)	Calcium intake > 1200 mg/day (*n* = 374)	*p*
Sex (females)	517 (95.0)	345 (93.2)	0.08
Age (years)	63.9 (25–37)	65.5 (37–94)	**0.03**
BMI (kg/m^2^)	25.5 (17.3–48.6)	24.8 (15.1–18.0)	0.015
Low BMD	236 (43.4)	177 (47.3)	0.24
Major fragility fractures	113 (20.8)	101 (27.6)	**0.028**
Other fragility fractures	92 (16.9)	66 (17.6)	0.77
Low BMD and/or major fragility fractures	274 (50.4)	204 (54.5)	0.21
Diabetes Mellitus	44 (8.1)	16 (4.3)	**0.022**
Hypercortisolism (endogenous or exogenous)	40 (7.3)	32 (8.6)	0.56
RA	76 (14)	50 (13.4)	0.79
Idiopathic hypercalciuria	28 (5.1)	12 (3.2)	0.16
PHPT	6 (1.1)	11 (2.9)	**0.042**
Nephrolithiasis	45 (8.3)	18 (4.8)	**0.042**
Adverse reaction to food	39 (7.2)	12 (3.2)	**0.01**
IBD	24 (4.4)	9 (2.4)	0.11
COPD	19 (3.5)	17 (4.5)	0.42

Data are expressed as median values (range) or absolute number (percentage)

Other fragility fractures: wrist, ribs and proximal humerus fractures; *BMI* body mass index, *RA* rheumatoid arthritis, *PHPT* primary hyperparathyroidism, *IBD* inflammatory bowel disease, *COPD* chronic obstructive pulmonary disease, *BMD* bone mineral density, *Low BMD* T-score at any site ≤  − 2.5 for postmenopausal women and men older than 50 or in the presence of Z-score at any site <  − 2.0 for premenopausal women and men younger than 50

### Prevalence and characteristics of the patients with a calcium intake < 700 mg/day

The Table 4 illustrates the comparisons between subjects with calcium intake < 700 mg/day and ≥ 700 mg/day. The patients with calcium intake < 700 mg/day showed an increased prevalence of idiopathic hypercalciuria, diabetes mellitus and of a personal history of adverse reaction to food compared to patients with calcium intake > 700 mg/day. Age, BMI, prevalence of low BMD, fragility fractures and other comorbidities (RA, endogenous or exogenous hypercortisolism, PHPT, nephrolithiasis, IBD and COPD) were comparable between the two groups.

**Table 4:** Comparisons between subjects with calcium intake < 700 mg/day and ≥ 700 mg/day

	Calcium intake < 700 mg/day (*n* = 544)	Calcium intake ≥ 700 mg/day (*n* = 1249)	*p*
Sex (females)	517 (95)	1157 (92.6)	0.06
Age (years)	65 (25–97)	65 (27–94)	0.17
BMI (kg/m^2^)	25.0 (17.3–48.6)	24.4 (14.2–44.9)	0.19
Low BMD osteoporosis	236 (43.4)	542 (43.4)	0.99
Major fragility fractures	113 (20.8)	255 (20.4)	0.86
Other fragility fractures	92 (16.9)	242 (19.4)	0.22
Low BMD and/or major fragility fractures	274 (50.4)	627 (50.2)	0.95
Diabetes Mellitus	44 (8.1)	66 (5.3)	**0.02**
Hypercortisolism (endogenous or exogenous)	40 (7.4)	97 (7.7)	0.72
RA	76 (14.0)	193 (15.5)	0.42
Idiopathic hypercalciuria	28 (5.1)	37 (3.0)	**0.02**
PHPT	6 (1.1)	24 (1.9)	0.21
Nephrolithiasis	45 (8.3)	87 (7.0)	0.33
Adverse reaction to food	39 (7.2)	51 (4.1)	**0.006**
IBD	24 (4.4)	41 (3.3)	0.24
COPD	19 (3.5)	52 (4.2)	0.5

Data are expressed as median values (range) or absolute number (percentage)

Other fragility fractures: wrist, ribs and proximal humerus fractures, *BMI* body mass index, *RA* rheumatoid arthritis, *PHPT* primary hyperparathyroidism, *IBD* inflammatory bowel disease, *COPD* chronic obstructive pulmonary disease, *BMD* bone mineral density, *Low BMD* T-score at any site ≤  − 2.5 for postmenopausal women and men older than 50 or in the presence of Z-score at any site <  − 2.0 for premenopausal women and men younger than 50

**Fig. 2:**
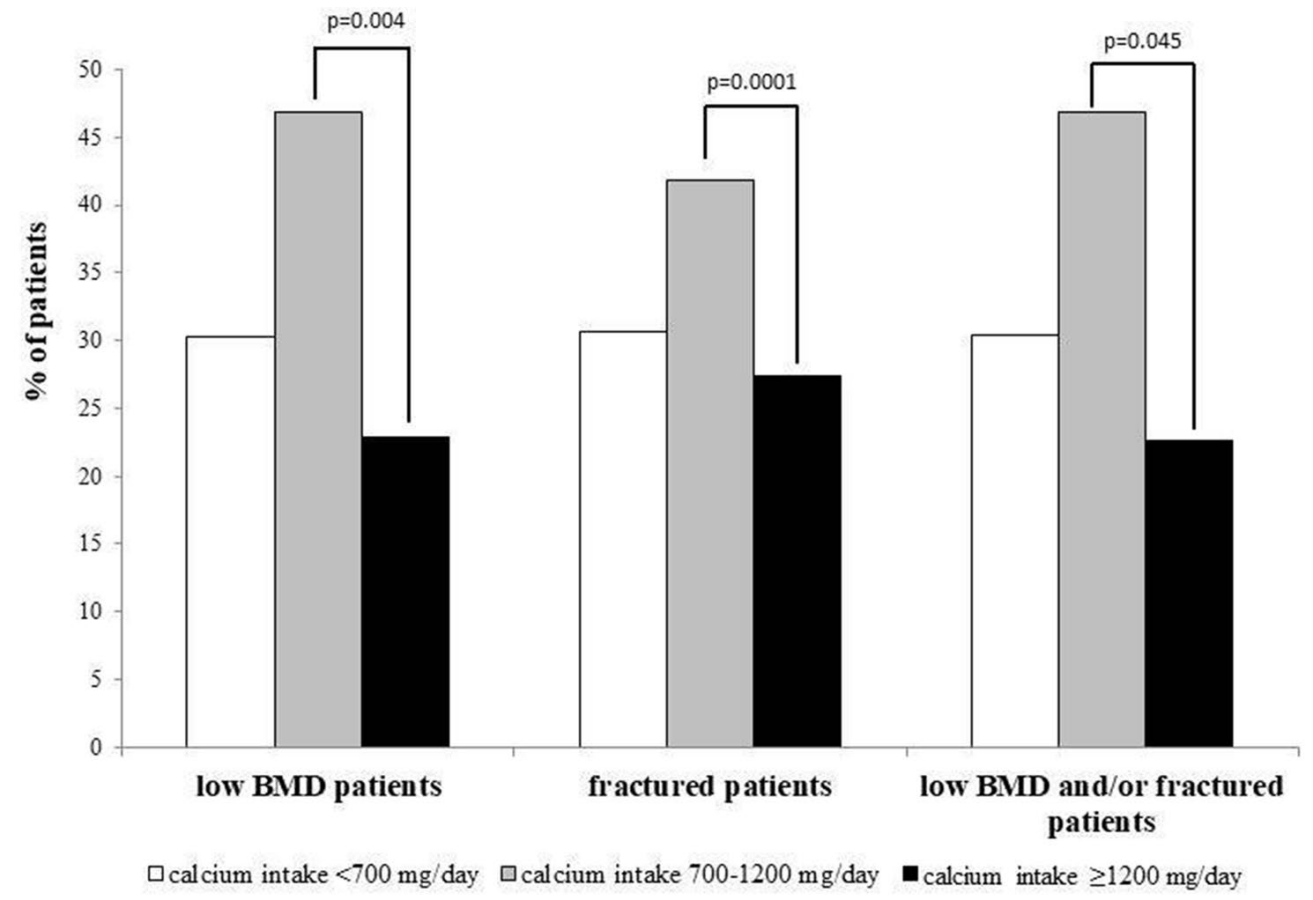
Prevalence of inadequate intermediate and adequate dietary calcium intake (< 700 mg/day, 700–1200 mg/day and ≥ 1200 mg/day, respectively), in a cohort of Italian subjects with possible osteoporosis. Data from 1793 individuals referred to outpatients clinics for osteoporosis of the Italian Society for Osteoporosis, Mineral Metabolism and Skeletal Diseases stratified on the basis of the presence of densitometric low bone mineral density (BMD) or fragility fractures or both. Among patients with low BMD (*n* = 778), 236 (30.3%), 365 (46.9%) and 177 (22.8%) had a dietary calcium intake < 700 mg/day, between 700 and 1200 mg/day and ≥ 1200 mg/day, respectively. Among patients with major fragility fractures (i.e., hip fracture and/or vertebral fractures) (*n* = 368), 113 (30.7%), 154 (41.8%) and 101 (27.5%) had a dietary calcium intake < 700 mg/day, between 700 and 1200 mg/day and ≥ 1200 mg/day, respectively. Among patients with low BMD and/or a major fragility fracture (*n* = 901), 274 (30.4%), 423 (46.9%) and 204 (22.7%) had a dietary calcium intake < 700 mg/day, between 700 and 1200 mg/day and ≥ 1200 mg/day, respectively

Interestingly, about one third of subjects with low BMD (236/778) and one third of subjects with fragility fractures (113/368) had a calcium intake < 700 mg/day (Fig. 2).

The logistic regression analysis showed that a calcium intake < 700 mg/day was independently associated with the female gender, a history of diabetes mellitus, food intolerance/allergy or a previous diagnosis of idiopathic hypercalciuria, regardless of age and BMI (Table 2).

### Prevalence and characteristics of patients with a calcium intake ≥ 1200 mg/day

The comparison between individuals with calcium intake < 1200 mg/day or ≥ 1200 mg/day is reported in Table 5. Patients with calcium intake > 1200 mg/day showed a lower prevalence of nephrolithiasis, but a higher prevalence of major fragility fractures and of history of previous PHPT compared to the patients with calcium intake < 1200 mg/day. Age, gender, BMI and prevalence of prior evidence of low BMD and other assessed comorbidities (RA, endogenous or exogenous hypercortisolism, IBD, food intolerance/allergy, COPD and diabetes) were comparable between the two groups. It is worth underlying that only 22.8% and 27.4% of low BMD and fractured subjects, respectively, had an adequate daily calcium intake (Fig. 2). A calcium intake ≥ 1200 mg/day was predicted by a low BMI, the absence of nephrolithiasis, a history of major fragility fractures and a previous diagnosis of PHPT, regardless of age and gender (Table 2).

**Table 5:** Comparison between individuals with calcium intake < 1200 mg/day or ≥ 1200 mg/day

	Calcium intake < 1200 mg/day (*n* = 1419)	Calcium intake ≥ 1200 mg/day (*n* = 374)	*p*
Sex (females)	1329 (93.7)	345 (92.2)	0.33
Age (years)	65 (25–97)	65 (37–94)	0.07
BMI (kg/m^2^)	24.7 (14.2–48.6)	24.2 (15.1–38.0)	0.06
Low BMD	601 (42.4)	177 (47.3)	0.08
Major fragility fractures	267 (18.8)	101 (27.0)	**0.001**
Other fragility fractures	268 (18.9)	66 (17.6)	0.58
Low BMD and/or major fragility fractures	697 (49.1)	204 (54.5)	0.06
Diabetes Mellitus	94 (6.6)	16 (4.3)	0.09
Hypercortisolism (endogenous or exogenous)	105 (7.4)	32 (8.6)	0.52
RA	219 (15.4)	50 (13.4)	0.32
Idiopathic hypercalciuria	53 (3.7)	12 (3.2)	0.63
PHPT	19 (1.3)	11 (2.9)	**0.032**
Nephrolithiasis	114 (8.0)	18 (4.8)	**0.034**
Adverse reaction to food	78 (5.5)	12 (3.2)	0.07
IBD	56 (3.9%)	9 (2.4)	0.16
COPD	54 (3.8)	17 (4.5)	0.51

Data are expressed as median values (range) or absolute number (percentage)

Other fragility fractures: wrist, ribs and proximal humerus fractures; *BMI* body mass index, *RA* rheumatoid arthritis, *PHPT* primary hyperparathyroidism, *IBD* inflammatory bowel disease, *COPD* chronic obstructive pulmonary disease, *BMD* Bone mineral density, *Low BMD* T-score at any site ≤  − 2.5 for postmenopausal women and men older than 50 or in the presence of Z-score at any site <  − 2.0 for premenopausal women and men younger than 50

### Calcium intake stratified for gender and presence of major fragility fracture and/or low BMD

The Table 6 shows the comparison of the clinical variables between males and females subjects and between fractured and not fractured patients. Male patients had older age and more frequently low BMD, major fragility fractures, RA, endogenous or exogenous hypercortisolism and COPD than female patients.

**Table 6:** Comparison of the clinical variables between males and females subjects and between patients with major fragility fracture and patients without major fragility fractures

	Females *N* = 1674	Males *N* = 119	Pts with major fragility fractures *N* = 368	Pts without major fragility fractures *N* = 1425
Sex (females)	–	–	334 (90.8)	1340 (94.0)^2^
Age (years)	64 (25–97)^1^	67 (27–89)^1^	71 (27–94)	63 (25–97)^3^
Adequate calcium intake (i.e., ≥ 1200 mg/day)	345 (20.6)	29 (24.4)	101 (27.4)^3^	273 (19.2)
Inadequate calcium intake (i.e., < 700 mg/day)	517 (30.9)	27 (22.7)	113 (30.7)	431 (30.2)
BMI (kg/m^2^)	25 (14.2–48.6)	26 (17.7–40.8)^1^	25 (16.5–48.6)	25 (14.2–48.2)
Low BMD	741 (95.2)	37 (31.1)^1^	245 (66.6)^3^	892 (37.4)
Major fragility fractures	334 (20.0)	34 (28.6)^2^	–	–
Other fragility fractures	314 (18.8)	20.0 (16.8)	105 (28.5)^3^	229 (16.1)
Diabetes Mellitus	102 (6.1)	8 (6.7)	25 (6.8)	85 (6.0)
Hypercortisolism (endogenous or exogenous)	22 (6.9)	22 (18.4)^3^	25 (6.8)	112 (7.8)
RA	234 (14.0)	35 (29.4)^3^	42 (11.4)	227 (15.9)
Idiopathic hypercalciuria	60 (3.6)	5 (4.2)	16 (4.3)	49 (3.4)
PHPT	0 (0.0)	30 (1.8)	5 (1.4)	8 (1.8)
Nephrolithiasis	120 (7.2)	12 (10.1)	28 (7.6)	104 (7.3)
Adverse reaction to food	80 (4.8)	10 (8.4)	14 (3.8)	76 (5.3)
IBD	60 (3.6%)	5 (4.2)	13 (3.5)	52 (3.6)
COPD	58 (3.5)	13 (10.9)^3^	21 (5.7)	50 (3.5)

Data are expressed as median values (range) or absolute number (percentage). ^1^* p* < 0.005, ^2^* p* < 0.05, ^3^* p* < 0.0001 males vs females or patients (pts) with major fragility fractures (i.e., hip fractures and/or vertebral fracture) vs patients without major fragility fractures patients. Other fragility fractures: wrist, ribs and proximal humerus fractures; *BMI* body mass index, *RA* rheumatoid arthritis, *PHPT* primary hyperparathyroidism, *IBD* inflammatory bowel disease, *COPD* chronic obstructive pulmonary disease, *BMD* bone mineral density, *Low BMD* T-score at any site ≤  − 2.5 for postmenopausal women and men older than 50 or in the presence of Z-score at any site <  − 2.0 for premenopausal women and men younger than 50

As compared with patients without major fragility fractures, patients with major fragility fractures were older and more often male and had more often a calcium intake > 1200 mg/day, low BMD and fragility fractures even at sites different from spine and femur.

Individuals with low BMD and/or fractures showed a higher calcium intake (949.9 ± 417.3 mg/day), were older (67.7 ± 10.3 years) and less frequently premenopausal (1.9%) than those without low BMD and fractures (907.5 ± 382.9 mg/day, *p* = 0.025; 61.3 ± 10.8 years, *p* < 0.0001; 8.7%, *p* < 0.0001, respectively.

Importantly, even excluding men and premenopausal females from the analyses, the results did not change (data not shown). As compared with premenopausal females, post-menopausal females had higher prevalence of low BMD and major fragility fractures and lower prevalence of calcium intake < 700 mg/day and adverse reaction to food, while BMI, prevalence of calcium intake > 1200 mg/day and of other fragility fractures, diabetes, hypercortisolism, RA, idiopathic hypercalciuria, PHPT, nephrolithiasis, IBD and COPD were comparable between the two groups (data not shown).

## Discussion

The recommended daily calcium intake in the adult population varies across countries and according to the different guidelines. Overall, a daily calcium intake of at least 1000–1200 mg/day is usually considered adequate [4, 6, 7, 21, 22]. Importantly, a quite recent systematic review shows that mean dietary calcium intakes fall below the recommended levels in many areas of the world [12], including Italy, where a survey on this topic performed in 2005–2006 revealed an average calcium intake in the adult population of 765 mg/day [13].

Although conducted in a different population (i.e., mainly postmenopausal women), the present study shows that, 15 years after that survey, the mean calcium intake in a cohort of Italian individuals referred for the evaluation of low BMD is still lower than recommended. Indeed, the mean daily calcium intake in the present study (about 875 mg), though better than in the past, is still insufficient, especially considering that these data were collected in mainly postmenopausal females and in a population at risk of low BMD. Indeed, it is well established that an adequate calcium and vitamin D intake is essential for bone health and that a low calcium intake is associated with an increased fracture risk [21, 23]. Our results are in agreement with those of previous studies showing a mean calcium intake lower than the recommended thresholds in osteoporotic populations, both in Europe [24] and in Italy [25].

It is worth noting that within our cohort almost one third of individuals with low BMD and/or fractures had an estimated calcium intake lower than 700 mg/day, the threshold below which the dietary calcium intake is considered to be associated with an increased risk of fracture and osteoporosis and below which a supplementation is considered mandatory [22] and less than a quarter of individuals with low BMD and/or fractures had an adequate daily calcium intake. Despite these findings, indicating an insufficient awareness of the importance of this nutritional issue for bone health, the prevalence of low BMD and previous major fragility fractures were higher in subjects of the third tertile than in those of the first and second tertiles of daily dietary calcium intake. This apparently surprising finding might be explained by the possibility that individuals with low BMD and/or fractures are more prone to increase the daily calcium intake. In keeping, in the present study individuals with low BMD and/or fractures were older than those without low BMD and/or fractures and the dietary calcium intake was directly associated with age. Accordingly, premenopausal females were more represented in the lowest tertile of daily calcium intake and had more frequently an inadequate calcium intake and less frequently an adequate calcium intake than post-menopausal women. Therefore, even if entirely speculative, this may suggest that the awareness of a low BMD and/or of a fragility fracture could have positively influenced the nutritional habits.

Moreover, individuals in the lowest tertile of daily calcium intake showed a higher prevalence of idiopathic hypercalciuria and of adverse food reactions (mainly lactose intolerance, to a lesser extent food allergy or autoimmune intolerance) than patients in the highest tertile. This result was also confirmed when we compared patients with calcium intake < 700 mg/day and > 700 mg/day and, in particular, these conditions were associated with an inadequate calcium intake independent of possible confounders, including the presence of low BMD and prevalent fragility fractures. These findings suggest that the presence of adverse food reactions and of hypercalciuria may have contributed to decrease the calcium intake. Indeed, despite the availability of lactose-reduced or lactose-free dairy products, lactose-intolerant individuals frequently avoid milk and derivatives which represent the main sources of dietary calcium and so are at risk of calcium inadequacy [26]. Likewise, although entirely speculative, it is conceivable that hypercalciuric subjects, possibly not appropriately informed, could have been worried of worsening the urinary calcium excretion by consuming dairy products. Conversely, it is known that an adequate dietary calcium intake is important even in hypercalciuric individuals to counterbalance the increased urinary loss and avoid a negative calcium balance [27]. Unfortunately, data regarding the type of hypercalciuria and the urinary calcium-to-creatinine/ratio are not available.

An inadequate calcium intake was also independently associated with the female gender and with the presence of diabetes mellitus. The first result, despite being consistent with previous data [12], acquires even more value considering that calcium requirements in women are generally higher than in men of the same age [22]. A history of diabetes mellitus could have negatively influenced calcium intake due to the need to follow not only a hypoglucidic, but also a cholesterol-lowering diet, which is a known factor risk for reduced calcium intake and low BMD [20]. On the other hand, it is not possible to exclude that the association between low calcium intake and prevalence of diabetes mellitus simply reflects a multi-morbidity condition and/or the socioeconomic status. However, in the present cohort, the BMI and the presence of diabetes did not influence the association between the presence of major fragility fracture and the calcium intake.

The comparison between patients with calcium intake < 700 mg/day and > 1200 mg/day showed that an adequate daily calcium intake was associated with a lower prevalence of nephrolithiasis, and a higher history of major fragility fractures and of a previous diagnosis of PHPT. The lower rate of nephrolithiasis in patients with adequate calcium intake is in keeping with the amount of data about the protective role against the lithogenic risk of the normocalcic diet [28].

We found an unexpected inverse association between calcium intake and prevalence of low BMD and of major fragility fractures (Table 1). Indeed, the prevalence of low BMD and of major fragility fractures were higher in patients included the I and II tertiles of calcium intake than in those in the III tertile (i.e., with the highest calcium intake). These differences are no longer present when comparing patients with calcium intake < 700 mg/day with the remaining subjects. This is explained by the fact that in the group of individuals with a calcium intake > 700 mg/day were comprised all subjects belonging to both the II tertile and the III tertile of calcium intake. In keeping, patients with adequate calcium intake (i.e., > 1200 mg/day) unexpectedly showed an increased prevalence of major fragility fractures. Similarly, the finding of an increased frequency of previous PHPT in patients with adequate calcium intake (i.e., > 1200 mg/day) as compared to those with a calcium intake < 1200 mg/day could be considered unexpected. In our opinion, these findings of a higher frequency of major fragility fractures and of a previous diagnosis of PHPT in patients with adequate calcium intake have a plausible explanation. Indeed, as discussed above, a previous fragility fracture and/or the finding of low BMD have probably encouraged subjects to increase their daily calcium intake. Likewise, a past diagnosis of PHPT and the subsequent post-surgical transient hypocalcemia may have contributed to increase the awareness of the importance of an adequate calcium intake in our patients.

Anyway, even though fractured patients have more likely an adequate calcium intake as compared to the not fractured ones, the proportion of fractured subjects with an adequate calcium intake does not exceed the 28%, thus indicating that over two-thirds of fractured patients had an inadequate calcium intake. Finally, the fact that hypercortisolism was not associated with a higher prevalence of adequate calcium intake confirms the still insufficient awareness in Italy of the importance of and adequate calcium intake for maintaining the skeletal health [29].

Overall, the independent predictors of a low calcium intake were the female gender, a history of diabetes mellitus, the presence of food intolerance/allergy and a previous diagnosis of idiopathic hypercalciuria, while age, BMI, the presence of low BMD and of major fragility fractures were not predictive of a low calcium intake. It must be observed that these predictors have been established by logistic regression analysis (classic statistics). However, osteoporosis is a multifactorial disease in which different factors and environments (i.e., dietary habits, age, BMI and comorbidities) interact in nonlinear biological mechanisms. Therefore, it is likely that this kind of problem may need a specific mathematical approach, such as the artificial neural networks, to be better understood, as suggested by previous studies in the setting of osteoporosis [19].

Thus, the lack of these statistic approach may be considered a first study limitation. The current study has, however, additional limitations. Firstly, the cross-sectional design permits to find associations but not to demonstrate a link of causality. Indeed, the recruitment of patients in referral centers for osteoporosis can have introduced a selection bias. Indeed, in some individuals the calcium intake assessed in this study really reflects the individual habits before the diagnosis of osteoporosis and/or the occurrence of a fragility fracture. However, many subjects could have been referred to these centers after the diagnosis of osteoporosis had already been made by their General Practitioners. Thus, it is not possible to discriminate whether these data reflect the attitude of the Italian General Practitioners in suggesting an adequate calcium intake in patients with at risk for fractures or the individual dietary habits. This possibility is reinforced by the finding that patients with middling dietary calcium intake (i.e., patients included in the II tertile, about the 50% of the sample) showed intermediate characteristics as compared with patients included in the I and III tertile as far as the prevalence of low BMD and/or fragility fractures was concerned. Secondly, we lack good tools for assessing dietary history and the food frequency questionnaires may not be accurate for estimating dietary intakes. In addition, the questionnaire assessed the dietary calcium intake at the moment of the study enrolment, and, therefore, could not have been informative of the dietary calcium intake in the past. Thirdly, the cohort is rather heterogeneous as it includes not only post-menopausal women, but even a small number of premenopausal women and men (5% and 6.6% of the entire cohort, respectively). As shown, male patients were older and affected by a more severe form of osteoporosis or by a more frequent secondary form than female patients and premenopausal female had more frequently a calcium intake < 700 mg/day and adverse reaction to food than postmenopausal ones. From looking at the analyses, it would appear that most men are screened because of comorbidities. This is what really happens in the routine clinical practice. As compared with females, men are generally considered at higher risk of secondary osteoporosis [30]. On the other hand, men are evaluated for possible osteoporosis only if they have a disease (or if they take drugs) that may impact on bone [31] or only after a fracture has occurred. In keeping, the male gender was found to be a predictor of a major fragility fracture in our sample. Therefore, in our opinion, the reduced awareness of the possible risk of osteoporosis in men could explain why most men have been screened only because of comorbidities. Finally, data about what type of fractures occurred and when these occurred and regarding the prevalence of vegan or vegetarian patients are not available.

These differences and lack of information should be taken into account when looking to the present data. It should be observed, however, that the inclusion of gender and the menopausal status in the logistic regression model did not influence the association between fractures and calcium intake. In addition, even excluding premenopausal females and males the results were confirmed. It is clear, however, that the inclusion of men and premenopausal females in such a study can be questionable. Nonetheless, we still believe that a “real life” study in all patients with a possible low BMD in Italy should be conducted on all subjects referred to our centers. This study design could have consented to obtain some data on the calcium intake in men and premenopausal women, that, nowadays, are lacking. However, we are aware that, given the low prevalence of men and premenopausal women, even the present study cannot give information regarding these populations. This is why in the present study the premenopausal status and male gender have be considered as possible covariates rather than objective of the study. Therefore, being over 90% of the subjects females and nearly 95% of them in postmenopausal status, this is essentially an analysis of postmenopausal females rather than a representative sample of the entire community. Given this large imbalance in the sample population, the results of this study cannot be generalized to the entire Italian population. Additionally, we are aware that the Italian recommendations are quite high at 1200 mg/day particularly considering that our patients have been referred for possible low BMD rather than for ascertained low BMD. However, considering that this study has been carried out in SIOMMMS referral Centers for Osteoporosis and Metabolic Bone Diseases and, as expected, 80% of patients were affected with osteoporosis and/or fragility fractures, we believe that the Italian Guidelines have been correctly adopted in this setting. Finally, we have to acknowledge also the following additional limitations of the study: (i) the lack of data on vitamin D status in our sample which could have been useful in the interpretation of the results; (ii) the fact that DXA machines for bone mineral density were not calibrated across participating canters; however, as these latter were all centers endorsed by SIOMMMS, the quality of their BMD assessment by DXA could be assumed as satisfactory; (iii) the lack of data regarding the presence of morphometric vertebral fractures, that could have led to possible incorrect categorization of patients with vertebral fractures.

The strengths of our study are related firstly to the large sample of patients included and to the “real life” design that consented us to describe a real picture of the current calcium intake in a population at risk for osteoporosis. Moreover, the exhaustive information obtained from participants gave us the possibility to describe particular populations at risk of inadequate calcium intake (i.e. patients with diabetes, idiopathic hypercalciuria and adverse reaction to food).

In conclusion, our data suggest that, to date, an inadequate calcium intake is still highly prevalent in a population with low BMD or at risk for this condition. Educational campaigns should be encouraged to correct the lack of knowledge about the safety and the benefits of an adequate calcium intake and, conversely, about the risks associated with a low calcium intake, particularly in osteoporotic or already fractured subjects. Currently, in Italy, several regional initiatives for consenting the General Practitioners to easily refer patients with severe osteoporosis and/or fragility fracture to a referral Centre for Osteoporosis are being setting up. These information regarding dietary calcium intake could be of help for optimizing the primary prevention of fragility fractures, starting by the General Practitioners themselves.

## Data Availability

The datasets generated during and/or analysed during the current study are available from the corresponding author on reasonable request.
